# Impact of fast-track management on adult cardiac surgery: clinical and hospital outcomes

**DOI:** 10.5935/0103-507X.20190059

**Published:** 2019

**Authors:** Cibelle Andrade Lima, Maria Karoline Ritchrmoc, Wagner Souza Leite, Diogo André Rodrigues Galdino Silva, Wildberg Alencar Lima, Shirley Lima Campos, Armele Dornelas de Andrade

**Affiliations:** 1 Universidade Federal do Rio Grande do Norte - Natal (RN), Brasil.; 2 Universidade Federal de Pernambuco - Recife (PE), Brasil.; 3 Real Hospital Português de Beneficência de Pernambuco - Recife (PE), Brasil.

**Keywords:** Airway extubation, Cardiac surgical procedures, Respiration, artificial, Vital capacity, Length of stay, Intensive care units

## Abstract

**Objective:**

To compare the impact of two fast-track strategies regarding the extubation time and removal of invasive mechanical ventilation in adults after cardiac surgery on clinical and hospital outcomes.

**Methods:**

This was a retrospective cohort study with patients undergoing cardiac surgery. Patients were classified according to the extubation time as the Control Group (extubated 6 hours after admission to the intensive care unit, with a maximum mechanical ventilation time of 18 hours), Group 1 (extubated in the operating room after surgery) and Group 2 (extubated within 6 hours after admission to the intensive care unit). The primary outcomes analyzed were vital capacity on the first postoperative day, length of hospital stay, and length of stay in the intensive care unit. The secondary outcomes were reintubation, hospital-acquired pneumonia, sepsis, and death.

**Results:**

For the 223 patients evaluated, the vital capacity was lower in Groups 1 and 2 compared to the Control (p = 0.000 and p = 0.046, respectively). The length of stay in the intensive care unit was significantly lower in Groups 1 and 2 compared to the Control (p = 0.009 and p = 0.000, respectively), whereas the length of hospital stay was lower in Group 1 compared to the Control (p = 0.014). There was an association between extubation in the operating room (Group 1) with reintubation (p = 0.025) and postoperative complications (p = 0.038).

**Conclusion:**

Patients undergoing fast-track management with extubation within 6 hours had shorter stays in the intensive care unit without increasing postoperative complications and death. Patients extubated in the operating room had a shorter hospital stay and a shorter stay in the intensive care unit but showed an increase in the frequency of reintubation and postoperative complications.

## INTRODUCTION

Cardiac surgery is a high-risk invasive procedure and is widely used as the treatment of choice for several cardiovascular diseases in different age groups.^(1,2)^ Given that it is an invasive procedure, it is common for the duration of mechanical ventilation in patients to vary from until the end of surgery to hours after admission to a recovery unit.

The concept of fast-track management in cardiac surgery is associated with the use of anesthetic drugs that allow the rapid discontinuation of mechanical ventilation in the postoperative period of patients undergoing cardiac surgery.^(3,4)^ Studies show that fast-track cardiac surgery management is associated with improved patient recovery, shorter lengths of stay in the intensive care unit (ICU), shorter hospital stays, and reduced health costs. In addition, some studies have shown that the procedure does not increase the risk of postoperative complications, mortality or reintubation rates.^(3,5-8)^

However, there is no standard definition of the fast-track strategy. Short-acting opioids are used in low doses to extubate the patient within a certain time, and this time is chosen arbitrarily by the surgical team because there are no physiological or pathological factors available to determine this time frame.^(7)^ Some studies have established a fast-track criterion for endotracheal tube (ETT) removal within 12,^(9)^ 10,^(10)^ 9,^(11)^ 8,^(12-14)^ 6,^(15-17)^ 7,^(18)^ 4^(5)^ or 2 hours^(19)^ after the surgery, with the patient in the ICU. In contrast, other protocols recommend the removal of the ETT while in the operating room, between 5 and 30 minutes after the surgery.^(20,21)^ This approach is called ultra-fast track.

There is little consistent data regarding the changes in lung mechanics and function in the postoperative period of fast-track cardiac surgery. Furthermore, it is important to more accurately assess the effects on lung mechanics and function, occurrence of reintubation, incidence of postoperative complications, length of ICU stay, and length of hospital stay in the different time intervals included in the fast-track concept to determine which extubation strategy promotes the best recovery of patients undergoing cardiac surgery.

The objective of this study was to evaluate the impact of fast-track management in the postoperative period of adult cardiac surgery on clinical and hospital outcomes at two different time points of ETT removal compared to a control group.

## METHODS

This was a retrospective cohort study conducted by collecting data from the medical records of patients admitted from March 2013 to June 2013 to the *Real Hospital Português de Beneficência* located in Pernambuco. It was approved by the Human Research Ethics Committee of the *Hospital Agamenon Magalhães* under opinion 475,522.

All patients undergoing cardiac surgery, aged between 20 and 80 years, extubated in the operating room or who remained under invasive mechanical ventilation for a period of up to 18 hours after admission to the ICU were included in the study.

Patients with surgical incisions other than sternotomy, history of thoracic surgery in the past year, need for intra-aortic balloon pump or extracorporeal membrane oxygenation, or with liver disease or chronic obstructive pulmonary disease were excluded.

The patients included were stratified into two groups of early extubation (fast-track) according to the time of ETT removal and a Control Group (CG). The CG was composed of patients who, despite undergoing similar anesthetic procedures as the other groups, were extubated after 6 hours from admission to the ICU, respecting the time limit of 18 hours. Group 1 (G1) consisted of patients undergoing ultra-fast track, with extubation occurring in the operating room 5 to 30 minutes after the end of surgery. Group 2 (G2) comprised patients extubated within 6 hours after admission to the ICU.

The following data were collected: age, gender, body mass index (BMI), type of surgery, and mortality risk for cardiac surgery according to the European System for Cardiac Operative Risk Evaluation II (EuroSCORE).^(22)^ The primary outcomes analyzed were vital capacity (VC) on the first postoperative day (maximum amount of air that can be expelled from the lungs after a maximum inhalation), length of ICU stay (days) and length of hospital stay (days). The secondary outcomes analyzed were reintubation (new endotracheal intubation after unsuccessful ETT removal), hospital-acquired pneumonia (HAP), sepsis, and death. HAP is defined as pneumonia that occurs 48 hours or more after hospital admission, and notification is made after medical diagnosis. The diagnosis of sepsis was made according to the hospital's protocol which defines it as an increase of two or more points on the Sequential Organ Failure Assessment (SOFA) after 24 hours.

### Patient management in the operating room and intensive care unit

The choice for fast track, as well as the selection of the time for ETT removal, was based on specific protocols of the surgical team and sedation depth. Anesthetic induction was similar in all three groups, using propofol, etomidate or thiopental, supplemented with midazolam, fentanyl or remifentanil; anesthesia was maintained with propofol or inhalational anesthetic (sevoflurane). Pancuronium or rocuronium was used for neuromuscular blockade. Thus, certain surgical-anesthetic teams opted for extubation in the operating room, while others maintained anesthesia so that extubation of the patient occurred after arrival in the ICU.

Patients who were going to be extubated in the ICU started the process of mechanical ventilation weaning after waking up. The spontaneous breathing trial was performed with the patient still under mechanical ventilation in pressure support mode with a pressure Delta of 7cmH_2_O and a positive end expiratory pressure (PEEP) of 5cmH_2_O for 30 minutes. Following extubation, all patients underwent noninvasive ventilation in the bilevel positive airway pressure (BiPAP) mode with IPAP (inspiratory positive airway pressure) between 10 and 16cmH_2_O to maintain the tidal volume at 6mL/kg and the EPAP (expiratory positive airway pressure) at between 5 and 6cmH_2_O. The patients' VC, regardless of the extubation time, was evaluated on the first postoperative day, with all patients already breathing spontaneously. The team performed the procedure through spirometry to determine the appropriate lung expansion therapy for each patient.

Standard intra- and postoperative monitoring included monitoring of radial artery pressure, central venous pressure, electrocardiogram, temperature, and peripheral oxygen saturation. Postoperative pain was treated with morphine and dipyrone to reduce the impact of pain on the patient's lung function.

### Statistical analysis

Statistical analysis was performed using the Statistical Package for Social Science (SPSS) software, version 20.0, considering p ≤ 0.05 as the statistical significance threshold for all tests.

The Kolmogorov-Smirnov test was performed to test the normality of the variables, followed by appropriate statistical treatment, according to the data distribution.

Qualitative variables were expressed as absolute and relative frequencies. The chi-squared test was used to assess the association of categorical variables and the incidence of HAP, sepsis, reintubation, nonrespiratory complications, and death between groups.

Normal quantitative variables were expressed as the mean ± standard deviation, and nonnormal variables were expressed as median and interquartile range (IQ) (25 - 75). The Kruskal-Wallis test with the post hoc t-test was used to characterize the sample and to analyze the VC variables, whereas the Kruskal-Wallis test with the post hoc Mann-Whitney test was used to analyze the lengths of ICU and hospital stay.

## RESULTS

A total of 223 patients grouped in G1 (n = 48), G2 (n = 78), and CG (n = 97) were included in the study, with a mean total time of ETT and total mechanical ventilation, from surgery to extubation, of 4 hours and 12 minutes, 8 hours and 43 minutes, and 15 hours and 48 minutes, respectively. Most patients were male, with a mean age of 57.45 years and overweight on average (27.61kg/m²). The stratification of mortality risk after cardiac surgery was evaluated by the EuroSCORE, with 88.1% of patients classified as low risk (zero - 2 points), 20% as medium risk (3 - 5 points) and 2.8% as high risk (> 6 points), with no difference between groups, as shown in table 1.

**Table 1:** Characterization of the sample population

	Total	Group 1 (n = 48)	Group 2 (n = 78)	Control Group (n = 97)	p value
Male gender*	140 (62.8)	35 (72.9)	47 (60.3)	58 (59.8)	
Age (years)†	57.45 ± 13.33	55.47 ± 13.7	55.42 ± 13.7	59.34 ± 11.1	0.058
BMI (kg/m^2^)†	27.61 ± 4.47	26.81 ± 4.1	28.7 ± 7.3	27.52 ± 4.7	0.280
EuroSCORE*					0.072
Low risk	192 (88.1)	45 (93.8)	65 (85.5)	82 (87.2)	
Medium risk	20 (9.2)	2 (4.2)	11 (14.5)	7 (7.4)	
High risk	6 (2.8)	1 (2.1)	0 (0)	5 (5.3)	
Surgery*					0.412
CABG	112 (50.2)	25 (52.1)	40 (51.3)	47 (48.5)	
MVR	39 (17.5)	6 (12.5)	13 (16.7)	20 (20.6)	
AVR	26 (11.7)	5 (10.4)	11 (14.1)	10 (10.3)	
CABG and/or MVR and/or AVR	22 (9.9)	3 (6.3)	6 (7.7)	13 (13.4)	
Other	24 (10.8)	9 (18.8)	8 (10.3)	7 (7.2)	
Drains*					0.501
Mediastinal	172 (77.1)	34 (70.8)	61 (78.2)	77 (79.4)	
Chest	5 (2.2)	1 (2.1)	3 (3.8)	1 (1)	
Both	45 (20.2)	13 (27.1)	13 (16.7)	19 (19.6)	
ECC*	152 (68.2)	35 (72.9)	55 (70.5)	62 (63.9)	

BMI - body mass index; EuroSCORE - European System for Cardiac Operative Risk Evaluation II; CABG - coronary artery bypass grafting; MVR - mitral valve replacement; AVR - aortic valve replacement; ECC - extracorporeal circulation.

* Variables compared by the chi-squared test.

† variables compared by the Kruskal-Wallis test. The results are expressed as n (%) or mean ± standard deviation.

Table 2 shows that there was an association between the occurrence of reintubation and the profile of the groups. There were only two cases of reintubation in G1, and both were due to respiratory depression and hypoventilation due to residual anesthetic effects. Thus, patients undergoing the ultra-fast track protocol, in which extubation was performed in the operating room, had a significantly higher occurrence of reintubation due to extubation failure.

**Table 2:** Association between the groups and occurrence of hospital-acquired pneumonia, sepsis, reintubation, postoperative complications, and death

	Group 1 (n = 48)	Group 2 (n = 78)	Control Group (n = 97)	p value*
HAP	2 (4.2)	2 (2.6)	7 (7.2)	0.355
Sepsis	1 (2.1)	1 (1.3)	4 (4.1)	0.492
Reintubation	2 (4.2)	0 (0)	0 (0)	0.025
Complications	7 (14.6)	5 (6.6)	3 (3.2)	0.038
Death	0 (0)	3 (3.8)	2 (1.94)	0.410

HAP - hospital-acquired pneumonia.

* Chi-squared test. The results are expressed as n (%).

There was also an association between the group profiles and the occurrence of nonrespiratory complications. G1 exhibited the highest frequency of complications, with 14.6% of cases due to cardiac tamponade (4), mediastinitis (1), pulmonary hypertension (1) or partial intestinal obstruction (1). In G2, the main complications were cardiac tamponade (1), acute renal failure (3), and pulmonary hypertension (1). The CG had the lowest frequency (3.2%) of complications, including acute renal failure (1), mediastinitis (1), and pulmonary hypertension (1).

There was no difference between the groups regarding the occurrence of HAP, sepsis, or number of deaths (Table 2).

**Figure 1:**
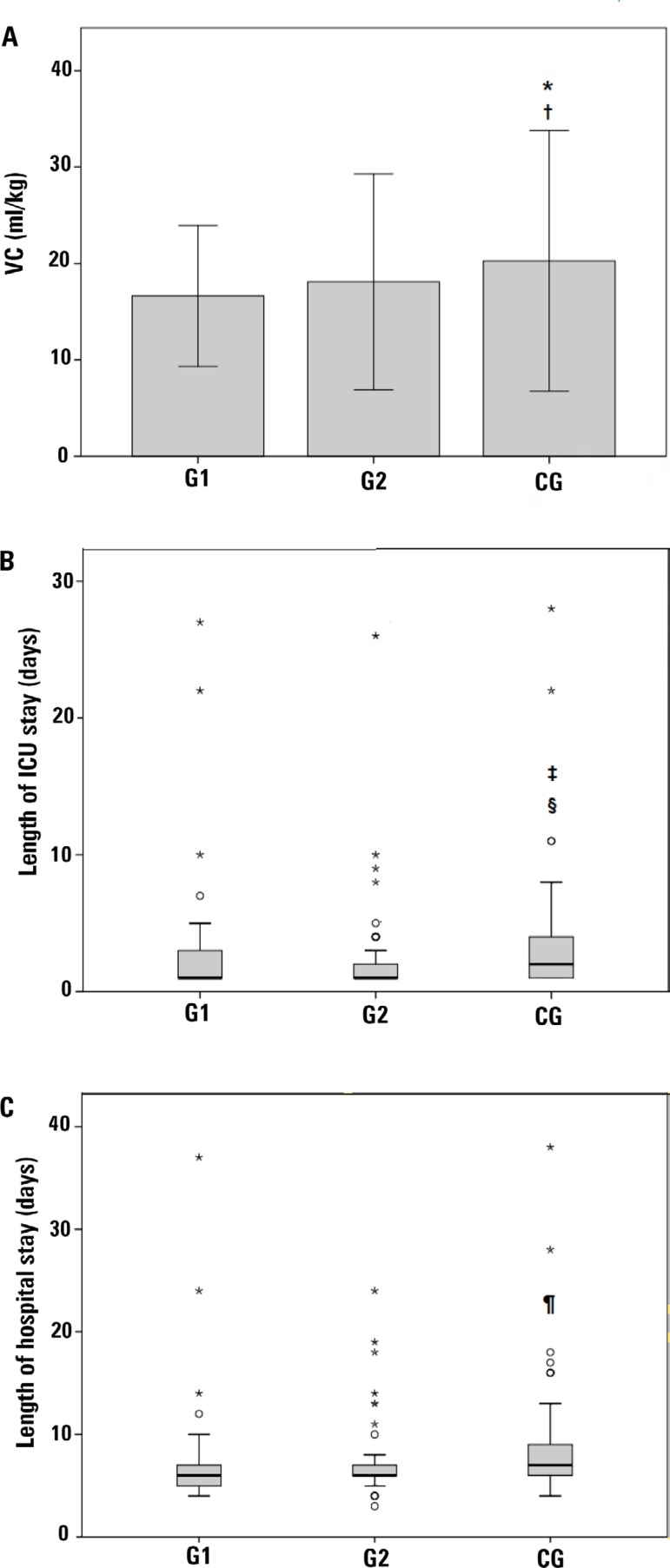
Intergroup analysis of vital capacity on the first postoperative day (A), length of stay in the intensive care unit (B), and length of hospital stay (C). VC - vital capacity; G1 - group 1; G2 - group 2; CG - control group; ICU - intensive care unit. * G1 *versus* CG (p = 0.000); ^†^ G2 *versus* CG (p = 0.046), Kruskal-Wallis test with post hoc T-test; ^‡^ G1 *versus* CG (p = 0.009); ^§^ G2 *versus* CG (p = 0.000), Kruskal-Wallis test with post hoc Mann-Whitney test; ^¶^ G1 *versus* CG (p = 0.014), Kruskal-Wallis test with post hoc Mann-Whitney test.

The VC on the first postoperative day and the length of ICU/hospital stay showed a significant difference between the groups (p = 0.010; p = 0.001; p = 0.004, respectively). The VC was significantly lower for G1 (16.87 ± 3.66; p < 0.001) and G2 (18.67 ± 5.59; p = 0.046) compared to the CG (21.03 ± 6.75), without significant differences between G1 and G2. The length of ICU stay was significantly lower in G1 compared to the CG (p = 0.009), with a median and IQ (25 - 75) of 1 (1 - 3) and 2 (1 - 4), respectively. The length of ICU stay was also lower in G2, with a median IQ (25 - 75) of 1 (1 - 2.25), compared to the CG (p = 0.000), and there was no difference between G1 and G2. Regarding the length of hospital stay, only G1 was significantly lower when compared to the CG (p = 0.014), with a median IQ (25 - 75) of 6 (5 - 7) and 7 (6 - 9), respectively. There was no difference in the length of hospital stay between G2, 6 (6 - 7), and the other groups (Figure 1).

## DISCUSSION

In cardiac surgery, patients conventionally receive anesthesia using high doses of opioids and would, consequently, be under invasive mechanical ventilation for a long period of time. In the early 1990s, the concept of fast-track procedures, which involves the use of low doses of opioids for achieving rapid extubation, was introduced to meet the growing demand for cardiac surgery. Currently, many surgical teams use an anesthesia protocol that involves the removal of the ETT in the operating room or within a few hours after the end of surgery.^(3,23,24)^

Many studies have demonstrated the benefits of using fast-track protocols in cardiac surgery. More recently, a systematic review of 28 clinical trials showed that fast-track management reduces the length of ICU stay without impacting mortality, postoperative complications (myocardial infarction and stroke), or reintubation within 24 hours when compared to the conventional protocol.^(23)^

Such evidence is in accordance with the results of this study regarding the analysis between G2 and CG. There was no difference in mortality, occurrence of postoperative complications, reintubation, HAP, sepsis, or length of hospital stay between these groups, but there was a reduction in the length of ICU stay in G2 when compared to the CG.

However, despite the recent evidence on the fast-track strategy presented by Wong et al.^(23)^, the systematic review revealed heterogeneity in the protocols of the studies included in the analysis, especially regarding the choice of time for ETT removal, grouping patients extubated in the operating room with patients extubated within 8 hours after their admission to the ICU. In our study, the analysis stratified patients according to the extubation time and identified some divergences from the previous investigation when analyzing the patients in G1 and the CG.

We found an association between the ultra-fast track protocol and the reintubation rate due to failure in the ETT removal. There were two (4.2%) cases of reintubation in G1 due to respiratory depression caused by residual narcosis. In addition, there was an association between the ultra-fast track and the occurrence of postoperative complications not related to the respiratory system. A similar result was also described by Montes et al.^(20)^ in a study where patients extubated in the operating room had a significantly higher incidence of reintubation (8%) compared to patients extubated in the ICU, and all cases were due to respiratory depression. The same study found a higher incidence of postoperative complications in the ultra-fast track group, but without statistical significance.

In contrast to the results found by Montes et al.^(20)^, our study showed a reduction in both the length of hospital stay and the length of ICU stay in G1 compared to the CG. Both clinical outcomes are important and desired because they reduce hospital costs and public health expenditures. Corroborating our findings, the studies performed by Cheng et al.^(15)^ and Michalopoulos et al.^(18)^ showed a reduction in the length of hospital stay with an early extubation protocol and, more specifically, the study conducted by Saad et al.^(21)^ showed a shorter length of ICU stay in patients undergoing extubation in the operating room.

Despite the association found for postoperative complications and reintubations in G1, there was no difference in mortality compared to the GC and G2. The low mortality rate found in the three groups is probably related to the low EuroSCORE of the population, as described by Shoji et al.,^(25)^ who reported that the lower the severity indices, the lower the possibility of death in this population.

The occurrence of reduced VC is common in the postoperative period of cardiac surgery due to a number of factors that include the surgical procedure itself, the use and time of extracorporeal circulation, sternotomy, and postoperative pain. Our results revealed a lower VC on the first postoperative day in the two fast-track groups when compared to the CG. However, this difference has little clinical impact because, in all cases, the VC had values below 25mL/kg, which reflects low lung expansion, capable of generating atelectasis and favoring the occurrence of respiratory complications. Pinheiro et al.^(26)^ noted that VC values lower than 25mL/kg predispose patients to the appearance of atelectasis and respiratory complications, and our results showed no difference in the occurrence of HAP between the groups.

This study had some limitations. Because it is a retrospective study based on the collection of data from medical records, there was no control regarding sample selection or randomization in the stratification of the groups, and the baseline characteristics of the patients could have influenced the results. It was also not possible to collect data on the time of extracorporeal circulation and the VC in the preoperative period because that is not a routine protocol in the hospital studied. Thus, evaluating the reduction in the VC based on individual baseline values was impossible. In addition, although there was no difference between the fast-track and control groups, the low EuroSCORE may have influenced the low incidence of the evaluated outcomes (HAP, sepsis, nonrespiratory complications, reintubation, and death).

Additionally, the lack of description in the medical records of an ultra-fast track operating room protocol can be indicative of a lack of standardization for the tracheal extubation procedure in this group of patients and, consequently, of a possible bias in the results. Thus, the results of the group that underwent such procedure should be evaluated with caution.

## CONCLUSION

This study demonstrated that in our sample the fast-track protocol with endotracheal tube removal in the intensive care unit is associated with a shorter stay in the intensive care unit without increasing the occurrence of postoperative complications, hospital-acquired pneumonia, sepsis, reintubations, or death compared with the conventional protocol. In turn, the ultra-fast track protocol reduces the length of stay in both the hospital and the intensive care unit; however, extubation of the patient in the operating room is associated with an increased occurrence of reintubation and postoperative complications.

The external validity of our results is limited, and therefore we emphasize that other studies should be conducted to determine the ideal time for removal of the endotracheal tube after cardiac surgery.
