# Erratum

**DOI:** 10.5935/0103-507X.20200050

**Published:** 2020

In the article **Impact of fast track on adult cardiac surgery: clinical and hospital outcomes**, with DOI number: 10.5935/0103-507X.20190059, published in the journal Revista Brasileira de Terapia Intensiva, 31(3):361-7, on page 361:

**Where it read:**

Maria Karoline Ritchrmoc

**Read:**

Maria Karoline Richtrmoc

